# The Desalting Process for Table Olives and Its Effect on Their Physicochemical Characteristics and Nutrient Mineral Content

**DOI:** 10.3390/foods12122307

**Published:** 2023-06-07

**Authors:** Antonio López-López, José María Moreno-Baquero, Antonio Garrido-Fernández

**Affiliations:** Instituto de la Grasa (IG), CSIC, Campus Universitario Pablo de Olavide, Edificio 46, Ctra. Utrera km 1, 41013 Sevilla, Spain; jose.moreno.baquero@gmail.com (J.M.M.-B.); garfer@cica.es (A.G.-F.)

**Keywords:** desalting kinetics, green plain table olives, green stuffed table olives, natural table olives, mineral leaching

## Abstract

The desalting process is critical for packaging table olives in brine with reduced NaCl or fortified mineral nutrients. In this study, the effect of desalting on the physicochemical characteristics and mineral content of green Manzanilla Spanish-style (plain and stuffed with pepper paste) and DOP *Aloreña de Málaga* table olives was investigated for the first time. The surface colour of the fruits turned slightly brownish, and the olives became somewhat softer. The lactic acid, the mineral macronutrients (mainly) and micronutrient contents decreased, while flesh moisture increased. The kinetic parameters of the minerals’ losses depended on the presentation, with the estimated values for plain olives being the lowest (slowest desalting). Overall, the desalting process resulted in slight quality damage and a moderated decrease in the mineral concentration in the flesh, leading to some product degradation. This study provides quantitative information on these changes that may affect the commercial value of the final products and offers information for viable designs.

## 1. Introduction

Fermented foods are prevalent worldwide, with table olives being the primary fermented fruit. The International Olive Council [1] reported a total production of 3.0 × 10^6^ tons during 2019/2020, according to the last balance adopted by the institution. The processing of table olives always involves fermentation/storage and final packaging in brine. The first step typically uses concentrations in brine around 8% NaCl [2], while the content in the second step may range between 5% and 6% NaCl for treated and natural olives or 10% for dehydrated olives [3]. Salty is one of the attributes included in the evaluation sheet for the sensory assessment of table olives [4].

Despite the thermal treatment of packaged products, which allows establishing NaCl levels according to good manufacturing practices, reducing its presence below 3% is challenging due to consumer perceptions of salt as a distinctive characteristic of table olives [5,6]. It is estimated that table olive consumers may intake from 6 × 10^4^ to 1.3 × 10^5^ tons of salt/season. However, consumers are increasingly concerned about salt intake [7]. The high-level Group on Diet, Physical Activity and Health in Europe adopted an EU Framework for national salt initiatives on 1 July 2008. The Member States agreed to create a common European Union Framework on voluntary national salt initiatives, with a minimum 16% salt reduction benchmark over 4 years for all food products. Bread was identified as the primary source of salt in the diet, followed by meat, meat products and dairy foods. The daily salt intake for Spanish men and women for the 2008–2012 period was estimated at 11.5 and 8.4 g salt/day, respectively [8], and has remained unchanged, with a current value of 9.7 g/day per person [9]. Following the European Union Framework, the *Agencia Española de Consumo*, *Seguridad Alimentaria y Nutrición* (AECOSAN) included these initiatives within the NAOS strategy, establishing as a first target 16% salt reduction [10], followed by meat products [8]. Table olives were not included in any of these EU initiatives due to their low consumption per capita [1]. However, the table olive sector is aware of its responsibility to reduce salt intake by consumers. Furthermore, according to market research [11], the global reduced-salt packaged foods market was expected to grow by 6.07% during 2018–2020. Mordor Intelligence [9] predicts an increase in low salt ingredients above 4.2% for 2020–2025. Then, there are opportunities for expanding the market for salt-reduced table olives.

Most investigations on reducing salt in table olives have focused on using salt mixtures during fermentation and storage processes [12,13,14,15,16], following the general trend in vegetable fermentations [17]. However, for robust processing and healthy risk prevention, the high levels of salt typically used during fermentation and bulk storage (usually over 8%) can hardly be reduced with the current state-of-the-art table olive technology. A more reasonable solution would be reducing salt in packaging, considering that stabilising the final products by thermal treatments (pasteurisation or sterilisation) is a widely extended practice at the industrial scale nowadays. To achieve this objective, the first step should be reducing the salt levels in the stored olives, regardless of the process. Per contra, this operation also implies risks due to the initial low salt of the desalting solution and the scarce investigation on this aspect. Additionally, the packaging could also be used to incorporate other salt mixtures. Moreover, reducing salt content and fortification of the final products with mineral nutrients could be an attractive innovation in table olives.

In short, reducing salt content before packaging table olives in low-salt or salt mixtures is compulsory for the survival of the table olive sector. The previous desalting would be an essential conditioning operation in such cases. This study used distributed model mass transfer to investigate the desalting process and its impact on the fruits’ physicochemical characteristics and nutrient mineral content. The study also analysed the diffusion kinetics of NaCl and other compounds of interest to table olive technology. The findings of this research can also help design appropriate processes for enhancing the mineral nutritional quality of table olives.

## 2. Materials and Methods

### 2.1. Olives

The plain (PGM) and red pepper paste-stuffed green Manzanilla (PPSGM) table olives for this experiment were provided by JOLCA SA (Huevar, Sevilla, Spain), and the traditional *Aloreña de Málaga* (TA) by Aceitunas Bravo SL (Alhaurín de la Torre, Málaga, Spain). The first two presentations underwent the typical green Spanish-style lactic acid fermentation processes, followed by grading (plain olives) or grading plus pitting and stuffing (stuffed olives). Natural *Aloreña de Málaga* was cracked and preserved in brine for 4 months in a cold room at 7 ± 1 °C. Therefore, all of the presentations were in the post-conditioning storage phase of equilibrium before industrial packaging. PGM and PPSGM had a size of 240 and 282 fruits/kg, respectively, while TA was in the EXTRA category. One hundred kilograms of each olive presentation was transported to the Instituto de la Grasa (CSIC) facilities and stored at 7 °C till use.

### 2.2. Desalting

The desalting process consisted of two phases. The first was used to estimate the time required for the desalting; it was performed with 1.228 (PGM), 1.000 (PPSGM), and 1.100 (TA) kg olives submerged into 2.000, 1.344, and 1.163 L desalting water, respectively. The ratios between olives and the desalting water were estimated, bearing in mind their NaCl concentrations in the storage brines and the expected 2.5% (*w*/*v*) NaCl in the equilibrium after desalting. The experiment was performed in a cold room at 8 ± 1 °C to prevent spoilage. Samples of olives and the desalting solutions (in the same proportion as in their respective containers) were taken over time and analysed for NaCl content. The results allowed a first approach for the estimation of the desalting period.

The second phase was performed at the pilot plant scale. In this case, 31.76 kg of PGM, 28.5 kg of PPSGM, and 42.5 kg of TA olives, introduced into 52.0, 40.6, and 45.2 L of desalting tap water, respectively, were used. The experiment was also carried out in a cold room at 8 ± 1 °C. Periodically, olives and desalting solutions (in the same proportions as in their respective containers) were taken and analysed for changes in moisture, lactic acid, Na, K, Ca, Mg, P, Fe, Cu, Mn, and Zn over time. In addition, the colour and firmness of the olives before and after desalting were measured. The results were used to estimate their diffusion kinetics and changes during desalting.

### 2.3. Physicochemical Analysis of Brines

The pH, NaCl, titratable acidity, and combined acidity in the brine were measured according to the procedures developed for controlling table olive fermentation [2]. Moisture in olive samples was estimated by drying their flesh in stainless steel plates to constant weight, using an electric oven at 106 ± 3 °C (DIGITHEAT 80L, J.P. SELECTA, Abrera, Spain). Lactic acid was determined following the procedure described by Montaño et al. [18]. A Waters 2695 Separations Module, a Waters 2414 Refractive Index Detector, and a computer with Empower two software (Waters Assoc., Milford, MA, USA) were employed using a Spherisorb ODS-2 (5 µm, 25 cm by 4 mm i.d., Teknokroma, Barcelona, Spain) column. Compounds were eluted with deionized water adjusted to pH 2.3 with phosphoric acid at 2.0 mL/min.

### 2.4. Instrumental Determination of Firmness

Firmness was measured objectively using a Kramer shear compression cell coupled to an Instron Universal Testing Machine (Canton, MA, USA). The cross-head speed was 200 mm/min. The firmness of the olives was expressed as the mean of 20 measurements, each of which was performed on pitted fruit (PGM), stuffed olives (PPSGM), or cracked olives without pits (TA). Shear compression force was expressed as N/g pitted olives.

### 2.5. Instrumental Measurement of Colour

Surface colour analyses were performed on olives using a BYK-Gardner Model 9000 Colour-view spectrophotometer equipped with computer software to calculate the CIE coordinates: L* (lightness), a* (negative values indicate green, while positive values indicate magenta), and b* (negative values indicate blue, and positive values indicate yellow). Interference by stray light was minimised by covering samples with a box that had a matte black interior. Each measurement was the mean of the values from 20 olives. The colour evolution was expressed as L*, a* and b* parameters or the hue angle (angular component) and chroma (radial component), both related to the polar representation [19]; also, the ratio −a*/b* was evaluated, although it is considered as a kind of internal standardisation [20].

In this work, *hue* (*h_ab_*) and C* (chroma) values were estimated from the equations:

(1) $$h_{ab}=\arctan\frac{b*}{a*}$$

(2) $$C*=\sqrt{a{*}^{2}+b{*}^{2}}$$

### 2.6. Mineral Analysis in the Pulp

All reagents were of analytical purity (Panreac, Barcelona, Spain). Hydrochloric acid (6N) solution was obtained by diluting concentrated HCl (Fluka, Buchs, Switzerland). The stock solution of Ca was obtained from Sigma Aldrich (St. Louis, MO, USA), while those of Na and K were purchased from PACISA (Madrid, Spain). The standard solutions were prepared by diluting the corresponding stock solutions and adding HCl in a concentration similar to that obtained in the sample solutions.

All glassware used to determine the mineral elements was immersed in 6N HCl overnight and then rinsed several times with distilled deionised water. The evolution of the desalting process was carried out by analysing triplicate samples from each method. The pulp of 100 g of each olive sample, separated from the pit manually when necessary, was mixed with a homogeniser Ultraturrax T25 (IKA-Labortecnik, Staufen, Germany). Then, 5 g of the resulting paste was weighed exactly in a quartz capsule. The capsule was put in a muffle oven model L9/11-B180 (Nabertherm GmbH, Lilienthal, Germany). The temperature was quickly brought to 100 °C and then increased slowly until ashing (550 °C), which was maintained for ≈8–10 h. The white-greyish ashes were slightly moistened, dissolved with three parts of 2 mL of 6N ultra-pure hydrochloric acid and gradually filtered through a filter paper into a 25 mL volumetric flask. Solubilisation was aided by slightly heating the capsule after every addition of hydrochloric acid. To ease filtration, a suction hood was used. After that, the filter was washed three times with 3 mL of deionised water and added to the volumetric flask. Finally, the flask was filled with deionised water until levelled. Simultaneously, a blank was prepared with only the reagents. Lanthanum chloride was added to the acid solutions of the ashes and the standard solutions in a final proportion of 1% (*w*/*v*) to prevent interference in Ca determination.

The mineral nutrients were determined by atomic absorption spectrophotometry using an air–acetylene flame. The measurements were made in a GBC model 932 AA (Dandenong, Australia) atomic absorption spectrometer equipped with three hollow multi-element cathode lamps for the analysis of Na and K (Photron, Narre Warren, Australia), Ca, Mg, and Zn (Photron, Narre Warren, Australia), and Cu and Mn (GBC, Dandenong, Australia). Instrumental conditions for each element were fixed according to the equipment manual [21]. P measurements were made in a Cary UV/Visible spectrometer model IE (Varian Australia, Mulgrave, Australia).

### 2.7. Kinetics of the Desalting Operation

In the laboratory desalting experiment, only the NaCl concentrations in olive flesh and desalting solutions were determined and plotted; their data were used to estimate the period required for reaching the 2.5% NaCl equilibrium but not for kinetic studies. On the contrary, the pilot experiment was devoted to studying the changes in the physicochemical characteristics and mineral release kinetics (described in this work).

Lumped or distributed models usually describe the mass or heat transfer in biological material interacting with its environment. The main difference between them regards the mass transfer mechanism. The first model ignores the internal resistance to the transfer, while the distributed models consider internal and external resistance [22]. Lumped models consider the system a single, homogeneous entity, assuming mass transfer occurs uniformly throughout. This simplifies the complexity of mass transfer by assuming uniform concentration or temperature profiles within the system. On the other hand, distributed models take into account the spatial distribution of mass transfer properties and assume that mass transfer occurs at different rates or concentrations in different regions of the system.

Typically, lumped models are expressed using exponential equations. These models consider that mass transfer is controlled by diffusion and are based on the hypothesis of Fick’s law [23]. However, Fick’s law assumes conditions such as uniform diffusion coefficient, specific geometries, and, most notably, steady-state concentration gradient, which can hardly be found in table olive desalting. On the contrary, these olives present a very diverse and heterogeneous structure, with various types of exposed diffusion (skin on the exterior and flesh in the interior when stuffed or cracked), leading to significantly different conditions. Furthermore, the steady-state concentration gradient principle is not fulfilled in table olive desalting since the concentration in the surrounding solution progressively increases and reaches equilibrium only at the end of the desalting process. In cucumber mass transfer, Fasina et al. [24] demonstrated that the lumped model type was appropriate to fit the exchange of diverse compounds (mainly sugars) in freshly brined fruits. In this work, the limited thickness of the epidermis and reduced flesh diameter, the lye treatment in the case of green Spanish style olives (PGM and PPSGM), and the concave internal surface provided by pitting (PPSGM) or cracking the olives (TA) enabled us to assume that the lumped models were also the most useful for expressing the mass transfer between the olive fruits and the surrounding desalting solution in all presentations. Furthermore, the great differences in shape and olive conditioning (pitting, stuffing or cracking) between these commercial products influence the choice of model able to be applicable regardless of the characteristics of the fruits. Therefore, we used the following lumped model:

(3) $$\ln\left(\frac{C-C_{eq}}{C_{0}-C_{eq}}\right)=-kt$$

where *C*_0_ stands for the initial concentration of the component in flesh (or desalting solution) at *t*_0_, initial time; *C*, the concentration in flesh (or desalting solution) of the component at time *t*; *C_eq_*, the concentration of the component at the equilibrium between flesh and desalting solution; *k*, component movement rate (expressed in *t*^−1^); and *t*, time. The whole model is quite similar to that of a formation/destruction first-order kinetic model and, like this, may also have an independent term. In addition, the equations are independent of the concentration units since the first term is a dimensionless ratio. Furthermore, the model is similar to that used by Azzouz et al. [25] to investigate the drying kinetics of grapes, who considered it as a simplification of the diffusion equation for a slab or sphere.

Alternatively, the equation (Equation (3)) may be written in the following format:

(4) $$C=C_{eq}-(C_{eq}-C_{0})e^{-kt}$$

From this, several other practical parameters of interest can be estimated:

(i) percentage change by unit of time (*C_trans_*)

(5) $$\%C_{trans}=\left(1-\frac{1}{e^{-kt}}\right)\;*\;100$$

(ii) time required to halve change:

(6) $$t_{50}=\frac{Ln2}{k}$$

where *Ln*2 stands for the natural log of 2 (two).

### 2.8. Data Analysis

A non-linear estimation procedure programmed in SigmaPlot 13 (Systat Software Inc., San José, CA, USA) calculated the kinetic parameters and standard errors. The same software also generated graphs and plots of the fitted models.

## 3. Results

### 3.1. Characterization of the Raw Material

The control of the desalting process requires proper characterisation of the raw material (fermented/stored olives). The average weights of individual olives from the studied presentations were 3.15 g (PPSGM), 4.32 g (PGM), and 4.52 g TA (Table 1). Therefore, the commercial fruits’ sizes were 318, 232, and 220 olives/kg, respectively. The equatorial diameter ranged from 1.56 (PPSGM) to 1.76 cm (PGM), while the height was between 1.77 (PPSGM) and 2.19 cm (PGM) (Table 1). Such dimensions mean that the olives are ovoids with only a difference of 0.09 cm between lengths of both axes (PPSGM) and 0.43 cm in the more unfavourable case (PGM) (Table 1). Then, the diffusion model for specific geometric forms is hardly applicable. However, the highest volume (4.83 cm^3^) corresponded to TA (Table 1), the intermediate to PGM (4.19 cm^3^), with the lowest being for PPSGM (3.15 cm^3^). Despite the dissimilarities, the densities of PGM and PPSGM were quite similar, but the differences were enough to separate incorrectly pitted olives from those properly stuffed just by flotation in an adequate brine [2]. Unexpectedly, the TA olives had the lowest values, possibly because of lower mineral content and higher fat. The flesh proportion is critical information for preparing the packaging brines. It means the pulp ratio over the whole olive, that is, the weight of the olives excluding the pit or the stuffing material. It is estimated by manually removing these olive components. The flesh proportion was the lowest for TA (about 83%), while it was very similar for PGM and PPSGM (referred to as the stuffing material in this case). Conversely, the pit proportions were complementary to 100 g and followed the opposite order to the flesh percentage. The moisture in TA was markedly lower than in PGM and PPSGM, which could be related to the absence of lye treatment in the first presentation since immersion in alkali usually expands the flesh structures [2]. The highest moisture in PPSGM (≈73%) was a combination of the contribution of the olives (86.9%) and the stuffing material (68.5%), which usually entraps abundant water [26].

In PGM and TA, the pit was 0.67–0.75 g and had a volume of ≈0.5 cm^3,^ while in PPSGM, the stuffing material was about 0.5 g, and its volume was slightly lower than that of the pit (0.34 cm^3^). As a result, the pits and stuffing material had higher densities than whole (plain) olives. The differences in density are used to separate properly pitted or stuffed olives from those retaining the pit or even a part of it.

Concerning the storage brine that covered the olives used for the assays (Table 2), there were noticeable differences between titratable acidity in Spanish-style Manzanilla (≈7 and ≈9 g/L for PGM and PPSGM, respectively) and cracked *Aloreña* (≈3.75 g/L, TA) due to the reduced lactic fermentation in the last product due to its storage conditions. The principal acidifying agent (Table 2) in lye-treated products (PGM and PPSGM) was lactic acid, which ranged from ≈10 to 18 g/L when determined by HPLC. In comparison, it was ≈1.76 g/L in cracked *Aloreña* (TA) because of the scarce acid produced during the natural fermentation/storage [27]. As a result, the pH of the green Spanish-style olives (PGM and PPSGM) was around 3.9, while that of the *Aloreña de Málaga* (TA) had a higher value (≈4.3). Such differences in pH between the two styles could hardly influence the desalting process but could be more favourable for *Aloreña de Málaga* browning.

Finally, the salt concentration in the brines of the fermented/stored products ranged from 73 to 94 g/L, with PGM (whole/plain lye-treated olives) having the highest concentration, PPSGM (lye-treated olives stuffed with red pepper paste) having the lowest, and *Aloreña* (TA, following the natural process) only slightly above the minimum salt level (PPSGM) (Table 2).

The desalting operation was designed considering the proportion of flesh in the olives, the moisture proportion in the olive flesh, and the NaCl concentration in the storage brine, which is usually assumed to be equilibrated with its concentration in the moisture of the olive flesh. In this work, the desalting operation aimed to reduce the concentration to a target level of 25 g/L in the olive flesh moisture (and the desalting solution in equilibrium). However, 50 g/L NaCl was also considered in the case of PGM. A shorter desalting process could be of interest for this presentation due to its slow Na diffusion, produced only through the external olive surface. In this case, the Na excess should be re-conditioned in the packaging.

The experiment was first performed at a laboratory scale (to estimate the approximate period required for desalting) and then at a pilot plant scale (to study the changes in the physicochemical characteristics and the kinetics of the mineral release). Both assays were performed with olives from the same batch of stored olives.

### 3.2. Estimation of the Approximate Period of Desalting

**Figure 1:**
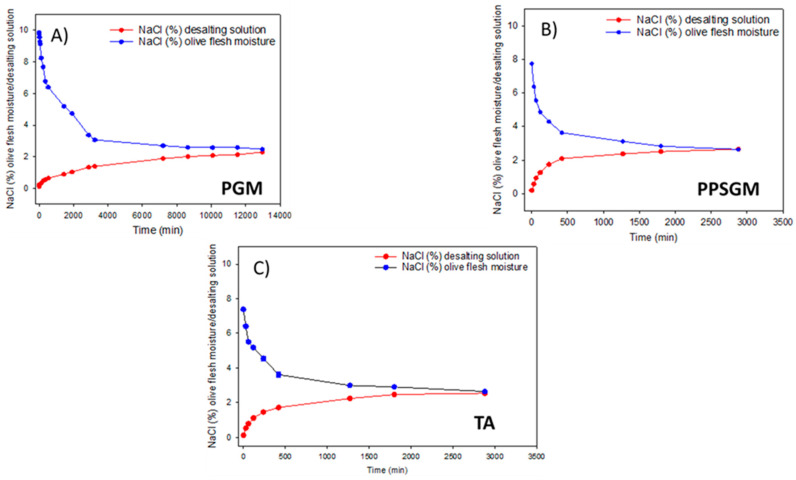
Changes in the NaCl concentrations over the desalting process. (**A**) Plain green Spanish-style Manzanilla table olives; (**B**) green Spanish-style Manzanilla table olives stuffed with red pepper paste; (**C**) traditional *Aloreña de Málaga* table olives.

This assay used the proportions of olives and water detailed in Section 2.2. The process showed progressive leaching of NaCl into the desalting solution, although with an important delay in PGM compared to PPSGM or TA (Figure 1A–C). In the first case, at a desalting temperature of 8 ± 1 °C, the equilibrium was reached in about 9 d (217 h), while in the case of stuffed (PPSGM) or cracked olives (TA), the time required was only approx. 2 d (50 h). The differences in the time needed to reach the equilibrium could be attributed to the internal surface available for the exchange in the case of PPSGM (the hole left by pitting, which, although filled with the stuffing material, allows contact with the surrounding solution) and TA (pit practically loosened after cracking). It should be emphasised that the presence of the stuffing material did not represent an obstacle to NaCl diffusion (olive flesh and stuffing material). The slower diffusion in plain olives should be related to the lower surface area for diffusion and the longer NaCl route from the inner part (close to the pit) to the olive surface that the elements must travel since the peel resistance is considerably reduced after the lye treatment.

In PPSGM (stuffed) and TA (cracked) olives, the leaching through the internal surface (pit loosened) markedly increased and accelerated the desalting. In cucumbers, the diffusion of NaCl took around 50 h to equilibrate [24], which is similar to the time required for PPSGM and TA but much lower than that for PGM, where diffusion depended on the presentation. However, diffusion of sugars and organic acids was slower and took around 100 h, depending on the size of the molecules. Furthermore, research has found that diffusion also depends on the anions present [24,28], with chloride having the highest mass transfer, while phosphate, sulphate or glutamate have significantly lower ones. A substantial proportion of the sodium in table olives can be present as lactate, which, in agreement with these authors, could decrease its mass transfer.

On the other hand, the temperature can also play an important role in Na diffusion [29], according to the Arrhenius equation [30,31]. Besides material structure and molecular components, the long desalting periods observed in this work can also be attributed to the low temperature (8 ± 1 °C) at which the operation should be performed since higher temperature results in more collisions against the tissues and, therefore, a faster rate of movement across them.

### 3.3. Pilot Plant Desalting

The assays at this level aimed to study the olive fruits’ concentration changes and the components’ diffusion kinetics in the different presentations. At this scale, the influence of sampling is minimised and allows scaling up the process for further packaging experiments using salt mixtures.

#### 3.3.1. Olive Fruit Changes Due to the Desalting Operation

The desalting operation, even when using a controlled temperature (8 ± 1 °C), impacted the fruits’ physicochemical characteristics and resulted in some degradation of olive quality. The changes were measured after reaching the desalting equilibrium. In plain olives (the slower desalting presentation), such evolution was estimated when the product reached 5% NaCl (Table 3), considering that, in this case, the desalting process could still be completed during packaging. A marked decrease in the colour index, luminance, b*, and chroma in PGM and TA was observed due to the apparent browning of the surface colour. However, the operation affected the PPSGM presentation less due to the antioxidant effect of the β-carotene in the pimento paste [26]. Additionally, a* values consistently decreased (olives were less greenish), particularly in the case of TA, where the degradation was 16%. The *hue* and −a*/b* were less sensitive to changes during desalting. In green Spanish-style Manzanilla olives fermented in a mixture of salts, colour changes were related to nutrient mineral concentrations [32], but in the desalting operation, the colour changes were more likely caused by the partial oxidation of the polyphenols since the surface colour appeared brownish at the end of the process.

The most relevant modifications observed due to desalting were related to firmness, with a marked decrease in the green Spanish-style presentations (PGM, ≈26% loss; and PPSGM, ≈13% decrease). In contrast, the lye-untreated fruits (TA) showed a limited effect (≈2% loss), indicating that the flesh in *Aloreña* retained its initial structure better. Moreover, the lactic acid concentration decreased sensibly, with 58, 62, and 69% reduction percentages for TA, PPSGM, and PGM, respectively. These losses are higher than those required to reach a concentration of around 0.5% in the equilibrium after further packaging. While this work was mainly focused on mineral leaching from the olive flesh, the parallel solubilisation of lactic acid was also of concern, and its kinetic diffusion was also studied.

The moisture content was also affected due to the olive flesh’s sponge-like structure. Depending on the surrounding liquid osmotic pressure, the flesh may release water (dehydration) or absorb it (hydration) [33]. As desalting occurs by submerging the stored olives in water, the olive flesh absorbs a marked proportion of moisture during the operation; the moisture absorption was greater in the PGM (≈10% increase), followed by TA (≈9%) and PPSGM (≈4%). The lye treatment in PGM had a limited effect on the re-hydration process since the moisture evolution in TA (without alkali treatment) showed an almost similar water absorption in the olive flesh (Table 3).

Conversely, the low absorption in PPSGM could be due to the high calcium concentration in this preparation, which may have strengthened the olive flesh and the red pepper paste, resulting in reduced olive flesh swelling when immersed in water. The strengthened olive flesh may have also hindered the release of lactic acid, as its loss (≈61%) was lower in PPSGM and its final concentration higher (6.9 g/L) than that observed in PGM (≈69%, 3.3 g/L, respectively) (Table 3). The behaviour could be compared to that observed in green Spanish-style Gordal fermentation in salt mixtures, where calcium delayed the release of sugars into the brine, resulting in reduced production of lactic acid [34]. The changes observed during the desalting process to reach 5% NaCl were relatively minor, indicating that prolonging the desalting period could be detrimental and may cause further significant degradation. Moreover, reducing the NaCl content from 5% to 2.5% NaCl requires a markedly more extended period due to the slower diffusion, as the salt gradient between the olive flesh and the surrounding solution becomes progressively less pronounced. Therefore, this work recommends desalting to 5% NaCl, which is a relatively short process (about 24 h) and causes minimal quality degradation.

#### 3.3.2. Salt and Mineral Nutrient Diffusion from Flesh into the Desalting Solution

The desalting process is a typical mass transfer operation studied in chemical engineering. Crank [23] provides a detailed analysis of this process. Several authors have studied the diffusion of Na into/from table olives. Drusas and Vagenas [35] developed a model to study Na diffusion into natural and lye-treated table olives, assuming the fruits were spherical and immersed in a large volume of solution. The experiment was conducted at 20 °C, and the concentration in the surrounding solution was almost constant (20 olives in 1 L solution). Sodium diffusion has been studied as a function of the lye concentration [36] and temperature [37]. Additionally, the same authors made several other contributions, including a simple method for researching the sodium diffusion in the epidermis of green olives [38], the determination of variables affecting sodium diffusion during debittering of green olives [39], and a study of sodium diffusion during *Aloreña* cultivar lye treatment [40]. The model was also used to investigate the diffusion of sugars and NaCl from the olives into the brine and NaCl in the opposite direction [41,42]. Later, a simple model of the diffusion phenomena (Na and Ca) taking place during the debittering process of green table olives was published [43]. More recently, Maldonado and Perez [44] developed a diffusion model based on olives for spherical and isotropic fruits, with a new function based on elliptical coordinates (olives are not perfect spheres but more frequently spheroids) also published [45]. While the diffusion of sodium during the debittering process of green Spanish-style olives is well-established, the desalting process is less well-known.

Furthermore, the components in the desalting solution undergo significant concentration changes during the operation. Sometimes, the olives have indefinable shapes (cracked olives), or it is not easy to assume model conditions. This section aims to provide a first approach to studying changes observed in various components during the actual conditions of the desalting process. It focuses on the effect on the nutritional properties of the olives, given that the operation also results in a sensible loss of mineral elements. In this regard, simple kinetic models used in other products such as cucumber [24], hawthorn [46], or grapes [25] have been demonstrated to be helpful and of practical application.

**Figure 2:**
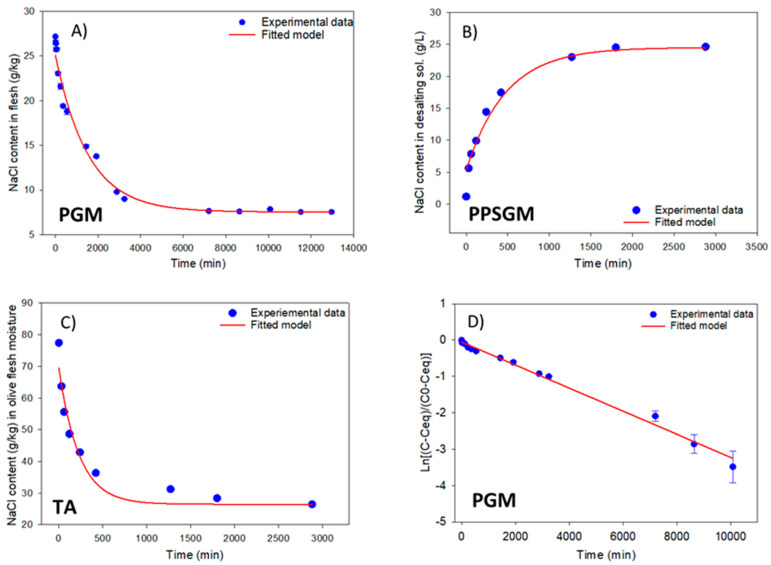
Changes in the NaCl concentrations, according to presentations and type of substrate. (**A**) Olive flesh of plain green Spanish-style Manzanilla table olives; (**B**) desalting solution used in green Spanish-style Manzanilla table olives stuffed with red pepper paste; (**C**) flesh moisture of traditional *Aloreña de Málaga* table olives; (**D**) olive flesh of plain green Spanish-style Manzanilla table olives.

The diffusion kinetics of the moisture, lactic acid and mineral nutrients into the desalting solution was followed by measuring the corresponding components in the olive flesh and the desalting solution over time. The characteristics of the process are described in detail in Materials and Methods (Section 2.2), and the features of the olives in the Results (Section 3.1). Examples of the NaCl mass transfer over time in various matrixes (flesh, desalting solution, and flesh moisture) are shown in Figure 2A–C. The dimensionless concentration evolution (left side) of Equation (3) was plotted against time for all components studied, and the model parameters were estimated by the minimum square sums, allowing for an ordinate equivalent to the change for t_0_ (Figure 2D), since important and rapid initial changes were frequently observed in several components. The fit was always highly significant, with the percentage of variance accounted for by the systematically high model (R^2^) and the low standard error of predictions (SE_est_) (Table 4). The ordinate (y_0_), the mass transfer coefficient (*k*), as well as their standard errors (SE) and *p*-values of the estimated kinetics parameters, are also reported (Table 4). The t_50_ and the proportion of change per hour were also calculated and are included in Table 4. As the mass transfer coefficient is independent of the units used for expressing the concentration, Equation (4) could fit the mass transfer of the various components studied (except flesh moisture) as a function of their concentration in the flesh, flesh moisture, or the desalting solution.

The mass transfer of all components, including moisture, into the flesh was lower in PGM than in PPSGM and TA (Table 4). This can be attributed to the fact that in PGM, only the external surface of the fruits is available for exchange, whereas PPSGM and TA have internal surfaces, without peel, exposed to exchange. Additionally, the diffusion in PGM may be obstructed by the firm consistency of the internal fraction of the flesh, which is not subjected to the lye action and covers approximately one-third of the inner flesh thickness. This hypothesis is supported by the observation that the time to reach t_50_ in PPSGM, which was treated with lye but pitted and with part of the inner flesh removed, was the lowest. Overall, the percentage change per hour (% ch/h) was the highest in TA, without lye treatment but with internal surface exposed to diffusion, while the t_50_ value was the lowest and increased for PPSGM (stuffed olives) and PGM (plain olives), respectively.

Regarding lactic acid, it exhibited the slowest exchange rate in PGM, followed by TA, partially due to the low acid concentration in this presentation; in contrast, lactic acid had an intermediate value in PPSGM, possibly because of its high calcium content, which strengthens the structure of the olive flesh and reduces the leakage of this element.

The mass transfer coefficients of the mineral nutrients varied among the different olive presentations. The lowest mass transfer rate was observed in PGM, which has the smallest surface area for exchange. However, PPSGM, which contained pepper paste and calcium, and TA, which did not undergo lye treatment, had similarly higher values. Na and K had higher diffusion values than divalent cations in PGM but followed a similar trend in the other preparations. In lye-treated olives (plain or stuffed), the mass transfer coefficients for calcium and phosphorous were slightly lower than those for the other minerals, but there were no appreciable differences in the case of TA. The t_50_ values for PGM were the longest, followed by those for PPSGM and TA, in agreement with the mass transfer coefficients. Conversely, the more rapid changes (% change/h) were inversely related to the coefficients. Based on the results of this pilot-scale experiment, desalting olives to 2.5% NaCl content would take 33, 10, and 6 h for PGM, PPSGM, and TA, respectively (Table 4). Therefore, this work provides practical details for the industrial application of the desalting process for the main green table olive presentations: whole and stuffed Spanish-style Manzanilla and cracked *Aloreña de Málaga*, the first Spanish POD. Additionally, the information is helpful for achieving not only the salt level recommended by the IOC [3] but any other concentration each industry deems suitable for its specific market. Moreover, the information may also be useful for any producer due to the similarities in cultivars and table olive processing worldwide.

#### 3.3.3. Effect of Desalting on Olive Mineral Nutrient Content

When it comes to nutritional labelling, it is required to express the mineral nutrients as their content in the edible portion of the final product. However, desalting table olives may lead to losses of various mineral nutrients, which could affect the overall nutritional value of the product (Table 5). In this study, desalting effectively reduced the sodium content in all three olive presentations, with the highest initial sodium content (PGM) experiencing the greatest reduction (from 27.2 to 7.5 g/kg flesh, 72%). The final sodium concentrations in PPSGM and TA were similar (7.3 g/kg flesh and 7.2 g/kg flesh, respectively) but had slightly lower losses (65% and 61%, respectively) due to their lower initial contents (21.2 g/kg and 18.6 g/kg flesh, respectively) (Table 5).

However, desalting also removed other mineral nutrients that were naturally present or partially incorporated during processing. Calcium, for instance, was high in the initial fermented/stored olives, ranging from 0.8 (PGM) to 2.8 g/kg flesh (PPSGM), which suggests intentional addition. Its leakage in PPSGM was the highest (45%), likely due to its use as a jellifying agent in the red pepper paste used to stuff the olives. In contrast, PGM (plain fermented olives, 19%) and TA (natural brined olives, 13%) had a low diffusion of calcium, likely due to its retention by the olive flesh structure, as noted by Jiménez et al. [47].

The remaining mineral elements in the three presentations were of natural origin, except for any possible small proportions incorporated from the salt or technological coadjuvant impurities. The most abundant of them during the whole desalting process was potassium (K), particularly in TA (due to direct storage in brine); however, a great proportion of K was lost (78, 65, and 66% in PGM, PPSGM, and TA, respectively). At the end of desalting, TA retained the highest content (0.843 g/kg flesh), while the lye-treated olives had low final concentrations (0.132 and 0.232 g/kg for PGM and PPSGM, respectively). The higher K abundance in PPSGM relative to plain olives after desalting should be due to the entrapment in the flesh and paste caused by the high concentration of calcium used to preserve the structure of stuffed olives. However, potassium is apparently not structurally important in processed olives and is significantly leached during desalting in most presentations (65–78%).

Despite being a divalent cation, magnesium did not follow the same trend as calcium but partially retained some of its characteristics since its behaviour was between that of calcium and potassium, with a percentage of loss slightly higher in the stuffed olives (Table 5). The final content of magnesium was far below the level required for its inclusion in nutritional labelling in the EU [48]. Finally, P was moderately retained in the flesh, and its leakage reached values of only between 30–40%, indicating that this element is also moderately retained by the flesh components. This circumstance may be one of the reasons for the good lactic fermentation of green olives since phosphorus is essential for the growth of lactic bacteria [2]. The micronutrients were also affected despite being firmly bonded to the olive flesh structure, resulting in unusually high iron content in the lye-treated olives. Its important release in PGM during desalting may indicate that its origin might be related to processing operations (size classification or transportation inside the plant). However, in the stuffed presentation, calcium also contributed to a certain degree of retention of other elements, as mentioned before.

Contrarily, in TA, the concentration was more consistent with those found in fresh olives due to limited manipulation during storage. Copper (Cu) and zinc (Zn) were present in low concentrations but were retained to a great extent (with low leakage), while manganese (Mn) was also retained in a higher proportion in the stuffed presentation. Desalting to 5% NaCl (PGM) caused lower effects on macro- and micronutrient minerals. In the first case, the release was intermediate, with respect to that observed for 2.5% NaCl. Meanwhile, most micronutrient minerals were sensibly leached during the 5% NaCl period. The further extension of the desalting period caused slight losses of these minerals due to their strong bonding to the flesh (Table 5).

## 4. Conclusions

The findings of this study indicate that desalting is a viable method for reducing salt content in fermented/stored table olives before packaging low-salt or mineral-fortified products. The research comprehensively analysed the desalting operation modelled for NaCl and other valuable components in table olive technology. Its impact on the physicochemical characteristics and the effect on the nutrient minerals were also studied. Unfortunately, desalting may cause, in addition to NaCl leakage, losses of other mineral nutrients and slight quality degradation. Fortification may increase the presence of the added minerals but not others. As a result, designing desalting operations for low-salt or mineral-fortified products necessitates preventing excessive loss of naturally occurring mineral nutrients and table olive quality. A suitable industrial-scale process could comprise reducing the NaCl level to only 5% during desalting and finalising the salt adjustment at the packaging stage. This approach would result in slightly lower mineral losses, better preservation of physicochemical characteristics, and a marked acceleration of the process.

## Figures and Tables

**Table 1:** Main characteristics (average, standard error in parentheses) of the raw material (storage products) from the different olive presentations used for the desalting experiment.

Characteristic	PGM	PPSGM	TA
Olive/stuffed product			
Weight (g)	4.32 (0.11)	3.15 (0.08)	4.52 (0.12)
Diameter (cm)	1.76 (0.01)	1.56 (0.02)	1.71 (0.02)
Height (cm)	2.19 (0.04)	1.77 (0.02)	1.97 (0.03)
Volume (cm^3^)	4.19 (0.15)	3.15 (0.05)	4.83 (0.13)
Density (g/mL)	1.03 (0.02)	0.99 (0.01)	0.94 (0.01)
Flesh proportion (%) *	84.58 (0.04)	84.20 (0.40)	83.07 (0.57)
Pit/stuffing material proportion (%) *	15.42 (0.40)	15.77 (0.03)	16.93 (0.57)
Moisture in the flesh/stuffed product	69.23 (0.28)	72.96 (0.11)	61.24 (0.09)
Pit/stuffing material			
Weight (g)	0.67 (0.02)	0.49 (0.01)	0.76 (0.02)
Volume (cm^3^)	0.49 (0.02)	0.34 (0.02)	0.46 (0.01)
Pit/stuffing material density (g/mL)	1.37 (0.04)	1.49 (0.05)	1.66 (0.03)
Pit/stuffing material proportion (%) *	15.42 (0.40)	15.77 (0.03)	16.93 (0.57)
Other characteristics			
Stuffing material moisture (%) *	--	68.49 (0.14)	--
Olive moisture without stuffing (%) *	--	86.90 (0.02)	--

Notes: PGM, plain green Spanish-style Manzanilla table olives; PPSGM, green Spanish-style Manzanilla table olives stuffed with red pepper paste; and TA, traditional *Aloreña de Málaga* table olives; *, % in *w*/*w*.

**Table 2:** Physicochemical characteristics (average, standard error in parentheses) of the brines of the raw material (storage products) from the different presentations used for the desalting experiment.

Brine Parameter	PGM	PPSGM	TA
pH	3.89 (0.03)	3.90 (0.02)	4.32 (0.03)
Titratable acidity (g/L)	7.07 (0.17)	9.20 (0.40)	3.75 (<0.001)
Combined acidity (mEq/L)	82.5 (1.8)	101.6 (3.4)	85.80 (1.56)
Lactic acid content (estimated) (g/L)	14.49 (0.31)	18.34 (0.09)	NA
Lactic acid content (HPLC) (g/L)	9.86 (0.07)	17.45 (0.29)	1.759 (0.018)
NaCl (g/L)	93.7 (0.2)	73.2 (0.3)	76.8 (0.06)

Notes: PGM, plain green Spanish-style Manzanilla table olives; PPSGM, green Spanish-style Manzanilla table olives stuffed with red pepper paste; and TA, traditional *Aloreña de Málaga* table olives; NA, not available.

**Table 3:** Changes in the main physicochemical characteristics (average, standard error in parentheses) of the fruits over the desalting process (final NaCl content in the flesh 2.5%, except in PGM, which included 5% NaCl) of the different presentations.

Parameter	PGM					PPSGM			TA		
	Stored	Desalted		Change		Stored	Desalted	Change	Stored	Desalted	Change
		5.0% NaCl	2.5% NaCl	5.0% NaCl	2.5% NaCl		2.5% NaCl	2.5% NaCl		2.5% NaCl	2.5% NaCl
Colour index	27.45 (0.36)	26.00 (0.12)	25.67 (1.61)	−5.2%	−6.2%	25.11 (0.35)	25.41 (0.38)	1.2%	38.94 (1.17)	31.45 (0.31)	−19.2%
L*	51.20 (0.18)	49.76 (0.06)	46.64 (1.44)	−2.8%	−8.8%	49.62 (0.06)	50.23 (0.11)	1.2%	56.11 (0.47)	51.31 (0.28)	−8.6%
a*	4.85 (0.13)	4.77 (0.006)	4.58 (0.06)	−1.6%	−5.6%	4.92 (0.07)	4.56 (0.17)	−7.3%	5.62 (0.06)	4.72 (0.07)	−16.0%
b*	35.90 (0.67)	32.06 (0.06)	31.91 (0.83)	−10.7%	−11.1%	32.97 (0.01)	33.09 (0.30)	0.4%	37.87 (0.18)	33.22 (0.08)	−12.3%
Chroma	36.23 (0.68)	32.41 (0.06)	32.23 (0.81)	−10.5%	−11.0%	33.34 (<0.01)	33.40 (0.31)	<0.1%	38.08 (0.16)	33.55 (0.08)	−11.9%
Hue	82.30 (0.22)	81.55 (0.17)	81.84 (0.05)	−0.9%	−0.6%	81.51 (0.12)	82.16 (0.21)	0.8%	81.51 (0.13)	81.91 (0.10)	0.5%
−a*/b*	−0.135 (0.002)	−0.148 (0.001)	−0.143 (0.005)	9.6%	5.9%	−0.149 (0.002)	−0.138 (0.004)	−7.4%	−0.149 (0.002)	−0.142 (0.002)	−4.7%
Firmness(N/g)	17.98 (0.17)	NA	13.23 (2.60)	NA	−26.4%	16.12 (5.10)	14.22 (4.50)	−12.7%	33.51 (0.51)	32.81 (1.81)	−2.1%
Moisture (%)	69.23 (0.28)	73.41 (0.09)	76.00 (0.04)	6.0%	9.8%	72.96 (0.50)	75.72 (0.07)	3.8%	61.24 (0.10)	66.44 (0.13)	8.5%
Lactic acid (g/L)	10.63 (0.01)	NA	3.30 (0.01)	NA	−69.0%	17.95 (0.34)	6.89 (0.10)	−61.6%	1.75 (0.013)	0.73 (0.01)	−58.4%

Notes: PGM, plain green Spanish-style Manzanilla table olives; PPSGM, green Spanish-style Manzanilla table olives stuffed with red pepper paste; and TA, traditional *Aloreña de Málaga* table olives; NA, not available.

**Table 4:** Kinetics parameters and standard errors of the desalting process from the original storage concentration to the 2.5% NaCl regarding the increase (moisture) or release (rest of compounds) in the olive flesh of the different presentations.

Compound/ Element	y_0_			*k* (min^−1^)			Fit Parameters		t_50_ (min)	% ch/h
	Estimate	SE	*p*-Value	Estimate	SE	*p*-Value	R^2^	SE_esti_		
Plain green Spanish-style Manzanilla table olives (PGM)										
Moisture (%)	−4.1 × 10^−1^	6.7 × 10^−2^	<0.0001	−5.0 × 10^−4^	2.2 × 10^−5^	<0.0001	0.953	3.7 × 10^−1^	1386	3.0
Lactic acid (g/kg)	−1.6 × 10^−1^	5.6 × 10^−2^	0.0094	−4.0 × 10^−4^	1.0 × 10^−5^	<0.0001	0.967	2.7 × 10^−1^	1733	2.4
NaCl (g/kg)	−1.1 × 10^−1^	2.9 × 10^−2^	0.0005	−7.0 × 10^−4^	1.1 × 10^−5^	<0.0001	0.995	1.6 × 10^−1^	990	3.7
K (g/kg)	−1.3 × 10^−1^	1.0 × 10^−1^	0.2105 **^◊^**	−7.0 × 10^−4^	1.9 × 10^−5^	<0.0001	0.949	6.2 × 10^−1^	990	4.1
Ca (g/kg)	−4.0 × 10^−1^	7.2 × 10^−2^	<0.0001	3.0 × 10^−4^	1.3 × 10^−5^	<0.0001	0.920	4.1 × 10^−1^	2310	1.8
Mg (g/kg)	−9.8 × 10^−2^	5.1 × 10^−2^	0.0595 **^◊^**	−4.0 × 10^−4^	9.1 × 10^−6^	<0.0001	0.976	2.9 × 10^−1^	1733	2.4
P (g/kg)	−1.3 × 10^−2^	4.4 × 10^−2^	0.7702 **^◊^**	−3.0 × 10^−4^	7.9 × 10^−6^	<0.0001	0.939	2.6 × 10^−1^	2310	1.8
Green Spanish-style Manzanilla table olives stuffed with red pepper paste (PPSGM)										
Moisture (%)	−1.3 × 10^−1^	7.4 × 10^−2^	0.0871 **^◊^**	−7.3 × 10^−3^	6.0 × 10^−4^	<0.0001	0.960	1.9 × 10^−1^	95	43.8
Lactic acid (g/kg)	3.4 × 10^−2^	6.0 × 10^−2^	<0.0001	−1.3 × 10^−3^	7.5 × 10^−5^	<0.0001	0.978	1.8 × 10^−1^	533	7.8
NaCl (g/kg)	−2.3 × 10^−1^	5.5 × 10^−2^	0.0002	−2.3 × 10^−3^	6.4 × 10^−5^	<0.0001	0.991	2.0 × 10^−1^	301	13.8
K (g/kg)	−4.2 × 10^−2^	1.5 × 10^−2^	0.0117	−3.4 × 10^−3^	7.1 × 10^−5^	<0.0001	0.997	0.4 × 10^−1^	204	20.4
Ca (g/kg)	−4.7 × 10^−2^	9.7 × 10^−2^	<0.0001	−2.0 × 10^−3^	1.0 × 10^−4^	<0.0001	0.963	3.7 × 10^−1^	347	12.0
Mg (g/kg)	−2.1 × 10^−1^	8.8 × 10^−2^	0.0250	2.8 × 10^−3^	1.0 × 10^−4^	<0.0001	0.983	3.4 × 10^−1^	248	16.5
P (g/kg)	4.8 × 10^−2^	6.8 × 10^−2^	0.4897 **^◊^**	1.6 × 10^−3^	8.5 × 10^−5^	<0.0001	0.969	2.6 × 10^−1^	433	9.59
Traditional *Aloreña de Málaga* table olives (TA)										
Moisture (%)	−2.6 × 10^−1^	7.0 × 10^−2^	0.0018	−4.3 × 10^−3^	3.0 × 10^−4^	<0.0001	0.953	2.1 × 10^−1^	161	25.8
Lactic acid (g/kg)	−2.2 × 10^−1^	6.8 × 10^−2^	0.0082	−3.4 × 10^−3^	3.0 × 10^−4^	<0.0001	0.956	1.7 × 10^−1^	204	20.4
NaCl (g/kg)	−2.2 × 10^−1^	4.4 × 10^−2^	0.0002	−3.7 × 10^−3^	2.0 × 10^−4^	<0.0001	0.954	1.3 × 10^−1^	187	22.0
K (g/kg)	−3.6 × 10^−1^	7.0 × 10^−2^	<0.0001	−3.3 × 10^−3^	3.0 × 10^−4^	<0.0001	0.926	2.1 × 10^−1^	210	19.8
Ca (g/kg)	−5.1 × 10^−2^	2.9 × 10^−2^	0.1031 **^◊^**	−3.6 × 10^−3^	2.0 × 10^−4^	<0.0001	0.974	0.7 × 10^−1^	193	21.6
Mg (g/kg)	2.7 × 10^−1^	5.5 × 10^−2^	0.0002	3.1 × 10^−3^	3.0 × 10^−4^	<0.0001	0.945	1.6 × 10^−1^	224	18.6
P (g/kg)	3.1 × 10^−1^	5.9 × 10^−2^	<0.0001	3.2 × 10^−3^	3.0 × 10^−4^	<0.0001	0.941	1.8 × 10^−1^	217	19.2

Notes: SE, standard error; R^2^, the proportion of variance explained by the model; SE_est_, standard error estimations; t_50_, time to reach half concentration; % ch/h, the percentage of change/hour; **^◊^**, not significant.

**Table 5:** Changes in the mineral nutrient content (mg/kg) in the flesh (unweighted average, standard error in parentheses) because of the different presentations’ desalting process. The final NaCl content in the flesh was 2.5%, except in PGM, which also included 5% NaCl.

Mineral Nutrient	PGM					PPSGM					TA		
	Content in the Stored Product	Conc. after Desalting		Released Amount (%)		Content in the Stored Product			Conc. after Desalting	Released Amount (%)	Content in the Stored Product	Conc. after Desalting	Released Amount (%)
		5.0% NaCl	2.5% NaCl	5.0% NaCl	2.5% NaCl	Stuffed Olives	Olives	Stuffing Material					
Na	27,185 (35)	15,011 (98)	7548 (65)	12,174 (50%)	19,637 (72%)	21,198 (36)	20,069 (126)	26,541 (126)	7340 (46)	13,858 (65%)	18,590 (223)	7205 (4)	11,385 (61%)
K	610 (3)	334 (9)	132 (2)	276 (45%)	478 (78%)	659 (8)	647 (8)	857 (8)	232 (1)	427 (65%)	2468 (11)	843 (1)	1625 (66%)
Ca	761 (1)	678 (10)	620 (2)	90 (12%)	141 (19%)	2813 (58)	2338 (31)	3226 (31)	1555 (11)	1258 (45%)	1189 (11)	1035 (1)	154 (13%)
Mg	140 (1)	105 (5)	62 (1)	35 (25%)	78.5 (56%)	194 (3)	180 (3)	233 (3)	77 (1)	118 (61%)	126 (1)	57 (1)	69 (55%)
P	129 (1)	NA	89 (1)	NA	40.1 (31%)	115 (2)	120 (2)	129 (2)	75 (1)	40.2 (35%)	189 (2)	119 (<1)	70 (37%)
Fe	29 (1)	18.4 (0.2)	16.7 (0.1)	10.23 (36%)	11.9 (42%)	29 (1)	27 (1)	23 (1)	25 (1)	4.2 (14%)	5.55 (0.05)	4.04 (0.03)	1.46 (27%)
Cu	2.09 (0.02)	1.55 (0.04)	1.51 (0.05)	0.54 (26%)	0.58 (28%)	1.30 (0.06)	1.69 (0.06)	1.03 (0.06)	1.08 (0.06)	0.22 (22%)	3.31 (0.03)	2.65 (0.04)	0.66 (20%)
Mn	1.07 (0.02)	0.63 (0.02)	0.40 (0.01)	0.44 (41.1%)	0.67 (62.6%)	0.28 (0.05)	NA	NA	0.27 (0.02)	0.10 (36%)	1.04 (0.06)	0.52 (0.01)	0.52 (50%)
Zn	3.95 (0.15)	2.19 (0.03)	NA	1.76 (44.5%)	NA	2.56 (0.10)	2.58 (0.09)	2.00 (0.09)	2.08 (0.11)	0.48 (19%)	4.03 (0.02)	2.57 (0.02)	1.46 (36%)

Notes: PGM, plain green Spanish-style Manzanilla table olives; PPSGM, green Spanish-style Manzanilla table olives stuffed with red pepper paste; TA, traditional *Aloreña de Málaga* table olives; NA, not available.

## Data Availability

Data is contained within the article.
